# Endless forms most stupid, icky, and small: The preponderance of noncharismatic invertebrates as integral to a biologically sound view of life

**DOI:** 10.1002/ece3.6892

**Published:** 2020-10-15

**Authors:** Jesse E. Czekanski‐Moir, Rebecca J. Rundell

**Affiliations:** ^1^ Department of Environmental and Forest Biology State University of New York College of Environmental Science and Forestry Syracuse NY USA

**Keywords:** animals, invertebrates, macroevolution, pedagogy, phylogenetic tree, *Xenoturbella*

## Abstract

Big, beautiful organisms are useful for biological education, increasing evolution literacy, and biodiversity conservation. But if educators gloss over the ubiquity of streamlined and miniaturized organisms, they unwittingly leave students and the public vulnerable to the idea that the primary evolutionary plot of every metazoan lineage is “progressive” and "favors" complexity. We show that simple, small, and intriguingly repulsive invertebrate animals provide a counterpoint to misconceptions about evolution. Our examples can be immediately deployed in biology courses and outreach. This context emphasizes that chordates are not the pinnacle of evolution. Rather, in the evolution of animals, miniaturization, trait loss, and lack of perfection are at least as frequent as their opposites. Teaching about invertebrate animals in a “tree thinking” context uproots evolution misconceptions (for students and the public alike), provides a mental scaffold for understanding all animals, and helps to cultivate future ambassadors and experts on these little‐known, weird, and fascinating taxa.

## Graphical abstract

There are many twists and turns in the tree of life. In this paper, we urge biology educators to include examples of evolution that remind students that evolution does not always result in organisms that are bigger, smarter, and/or more beautiful.

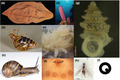

## 1 INTRODUCTION

“Thus, from the war of nature, from famine and death, the most exalted object which we are capable of conceiving, namely, the production of the higher animals, directly follows. There is grandeur in this view of life, with its several powers, having been originally breathed into a few forms or into one; and that, whilst this planet has gone cycling on according to the fixed law of gravity, from so simple a beginning endless forms most beautiful and most wonderful have been, and are being, evolved” (Darwin, 1859).

Understanding evolution is a cornerstone of scientific literacy (Allmon, 2009) that involves teaching students and members of the public that “endless forms” are genealogically related and that change occurs over time. Canonical illustrations (Table 1) of evolutionary history evoke grandeur and beauty: Hominins evolved bigger brains, whales and horses evolved from much smaller ancestors, and peacocks evolved impressive mating displays. Given these big, beautiful, and backboned examples of evolution, a naïve person would not be faulted for thinking that evolutionary change tends to make lineages smarter, bigger, and more beautiful through time. Even savvy students and educators can accidentally frame evolution as “progressive” (Rigato & Minelli, 2013; Werth, 2012). This idea permeates common evolution misconceptions, including characterizing organisms as climbing a ladder of progress, increasing in complexity over time, as “higher” or “lower,” “more evolved,” or “less evolved” (Allmon, 2009; Werth, 2012).

**Table 1:** Summary of commonly used examples in undergraduate‐level evolution courses, some critiques of the unintentional consequences of focusing on those examples, and suggested alternative examples (also discussed throughout the body of this article)

Typical example	Illustrates	Critique	Underutilized example	Illustrates
Hominin evolution	Changes in morphology through evolutionary time; our origin story	Certainly important to teach, but in isolation might leave students with the impression that organisms tend to become larger and smarter through time	Barnacles and tapeworms	Very successful, but almost certainly have more simple brains than their ancestors in the early Paleozoic
Horse evolution	How tooth and limbs have changed to become better adapted to different environments	Similar to hominin evolution: but also shows smaller ancestors and few terminal taxa that are larger than their Eocene ancestors	Meiofauna and miniaturized parasitoid insects	There are many lineages that have become smaller as an adaptation related to exploiting novel resources.
Evolution of peacock tails	Complex, beautiful traits can arise from sexual selection	Evolution does not always lead to increases in complexity or esthetic beauty	Placozoans, xenocoelomorphs, sponges, ostracodes	While arguably beautiful to the right beholder, these lineages have remained simple, or become more simple over long stretches of time
The "march of the phyla"	Presenting organismal phyla in a sequence from "simple" to more "complex" facilitates the increasing difficulty and specificity of dissections and morphological terminology	Although it may seem expedient, starting a course with sponges (or unicellular organisms), and ending with the Craniata, unintentionally echoes *Scala Naturae*‐style hierarchical views of life	Mix up the order in which phyla are introduced and/or complete “deuterostome” phyla (echinoderms, chordates) before tackling “protostomes”	Can better understand modern animal phylogeny as well as the phylogenetic consequences of the relatively sudden and simultaneous emergence of animal phyla; countering expectation can bring students to attention

**Figure 1:**
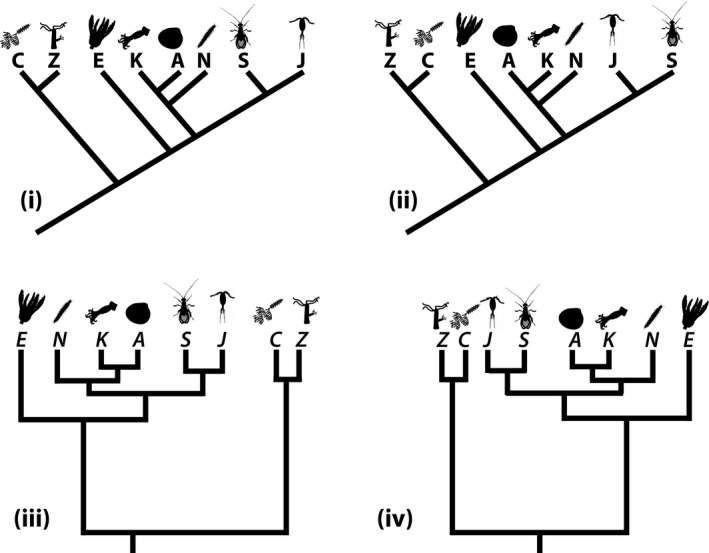
Many examples that are similar to this figure have been printed in previous publications about the pedagogy of tree thinking, but many figures contain three or four branch tips, and thus obscure how truly challenging it is, even for some experienced evolutionary biologists, to check to see whether two phylogenies illustrate equivalent topology, as all four of these do. Although examples (i) and (ii) are in a phylogeny format sometimes referred to as a “ladder phylogeny,” the overly simplistic “ladder thinking” we are trying to avoid would be someone who sees the first phylogeny and interprets it as C is the ancestor of Z, which is the ancestor of E, and so on. Displaying moderately complicated phylogenies in multiple formats (and perhaps including a phylogeny that is *not* topologically equivalent, and asking the students to identify the discrepancy) is a good way to help reinforce the proper interpretation of phylogenies. Image silhouette credits: A: Scott Hartman; C: Gareth Monger; E: Lauren Sumner‐Rooney; J: Kamil S Jaron; K: Becky Barnes; N: Scott Hartman; S: Joanna Wolfe; Z: Steve Haddock For a key to the taxonomy and full attributions of the *phylopic* silhouettes, as well as some suggestions for activities to do with this phylogeny, see Appendix S2 and Figure S1

Progress is insidious in the common conception of evolution in part because of our tendency toward teleology and seeing all change as improvement (Werth, 2012). This perception of evolution as progress might also stem from the relative attention paid to macroevolution in evolution education versus more thorough treatment of microevolution (Catley et al., 2012). Depictions of progressive and linear change from “simpler” to “more complex” species, not much different from the *scala naturae* or Linnaeus's classification of animals, are still common in modern textbooks (Catley et al., 2012) and even peer‐reviewed science literature (Werth, 2012). This ladder‐like view of evolution runs counter to tree thinking, which involves developing the skills to interpret information about evolutionary relationships using phylogenies, and make inferences using those phylogenies (Catley et al., 2012) (Figure 1).

**Figure 2:**
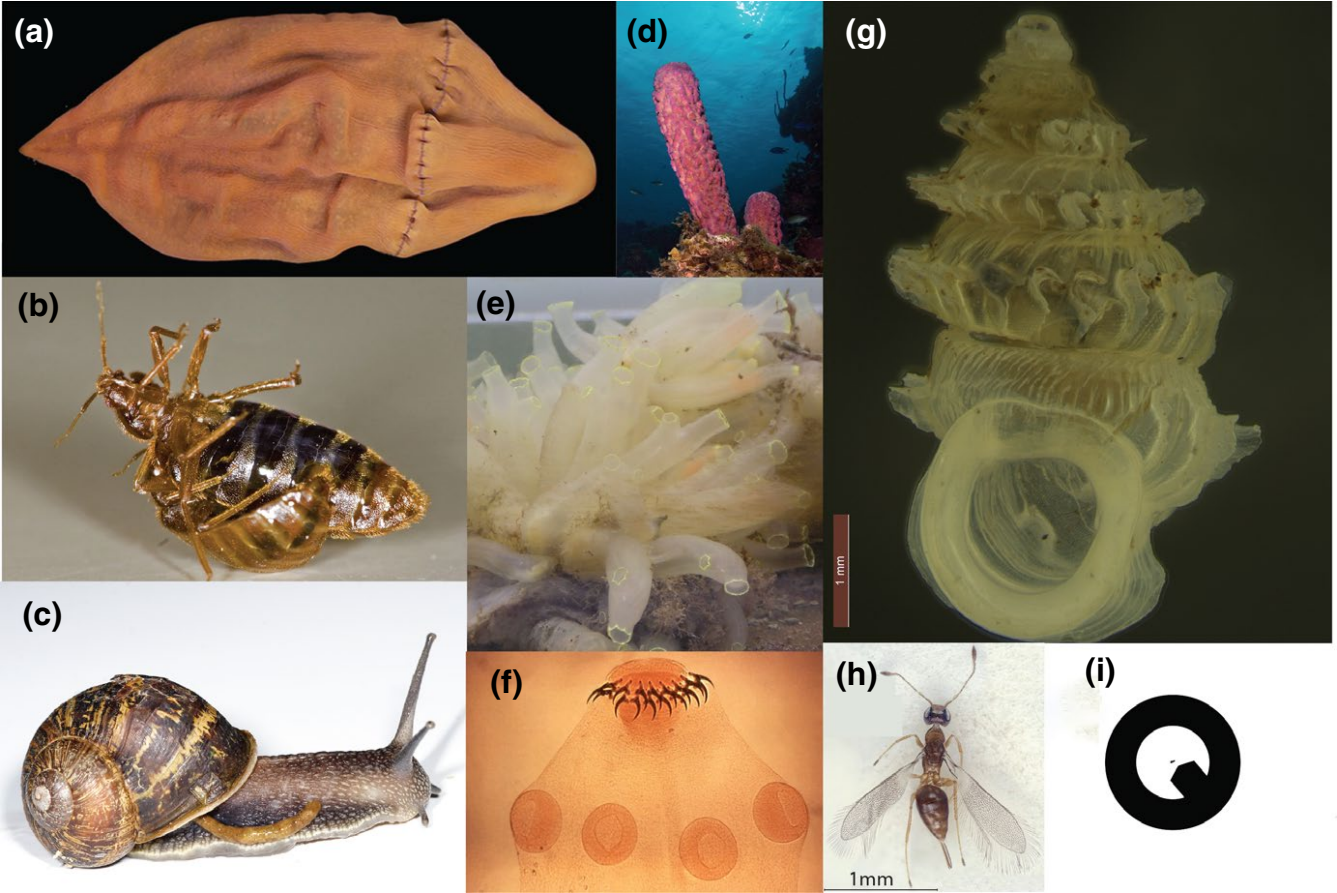
Representative forms most icky (a, b, c), stupid (d, e, f), and small (g, h, i). (a) The enigmatic *Xenoturbella churro*. (b) Bedbugs engaged in traumatic insemination. (c) The common garden snail exhibiting the less‐than‐ideal consequence of torsion: a tendency to defecate on its body. (d) Sponges, like this *Aplysina acheri*, are beautiful and extremely efficient filterers, but not particularly intelligent. (e) These adult sessile suspension feeding tunicates *Ciona intestinalis* (Urochordata) actually have more neurons than their free‐living tadpole larvae, but still fewer neurons than their craniate vertebrate relatives. (f) Cestodes such as *Taenia solium* have a scolex, often with hooks or suckers (both, in this case) for attachment in the host's intestine. The scolex is developmentally the anterior of the body, and does often have ganglia, but there are usually only around 400 neurons or fewer, an order of magnitude fewer than some of the much smaller, free‐living members of the phylum. Parts a*–*f are not to scale, but images g*–*i are to the same scale (scale bars in g and h are 1 mm). (g) is a diplommatinid land snail representative of the most diverse and abundant land snail family in the Republic of Palau. (h) is a member of the hymenopteran family Mymaridae, which contains some of the smallest species of insects described (note the ptilopterous (fringed with hairs) wings, characteristic of many of the smallest flying insects). (i) is what a 50 µm gastrotrich would look like at the same scale as the diplommatinid and the wasp (the tiny dot in the middle of the circle). Image credits (all from Wikimedia Commons except JCM images): a: Greg Rouse, Scripps Oceanography; b: Rickard Ignell, Swedish University of Agricultural Sciences; c: Fir0002/Flagstaffotos; d: Nick Hobgood; e: Perezoso; f: Centers for Disease Control and Prevention, 1986; g: JCM; h:(Huber, 2013); i: JCM, adapted from Rundell & Leander, 2010

Tree thinking has the potential to transform students' understanding of macroevolution (Catley et al., 2012; O’Hara, 1997), but we think instructors' choices of examples also matter. Using tree thinking in tandem with familiar vertebrate examples weakens this method's ability to address the “evolution as progress” fallacy. This is in part because vertebrates, mammals in particular, are presumed to be at the “top” of the tree and most complex. Although evolution lacks preordained direction, focus on vertebrates means we unwittingly suggest it does, by missing the diverse but often obscure sessile suspension feeders (i.e., animals that remove suspended food particles from water by trapping, capture, or filtration generally involving mucus (Brusca et al., 2016)), endoparasites, and examples of extreme miniaturization, all of which occur on multiple branches of the mostly invertebrate animal tree of life. These animals are not exceptions in evolution: They are closer to the rule. Darwin's “endless forms” are actually less grand than we often presume. In fact, among animals there is actually a preponderance of species that most would not recognize as being very smart, with small body sizes, and rather “icky,” off‐putting lifestyles (Figure 2). Among animals, neuron number can decrease through time, tiny animals can diversify and dominate (both phylogenetically and ecologically), and grotesque features, much less beautiful than a peacock's plumage, evolve and persist through time. We seek to upend students' (and the public's) deeply held notion of evolution as progress by using a tree‐based and organismal biology approach, with invertebrate animals as the chief exemplars. Phylogenetic reconstructions that inform this approach reveal evolution as multiply and extraordinarily branched (often likened to a “bush” (Gould, 1986)), not a ladder, whose branches show neither progress nor perfection. The characteristics of invertebrate animals studding this phylogenetic tree are obscure to most people. Combining phylogenetic evidence with knowledge about this majority share of animals can reinvigorate biology teaching and outreach by revealing evolution for what it is: proliferation among unexpected dead ends, rampant evolutionary reversals, stunning stretches absent of grandeur, imperfect but serviceable solutions, and a dominance of the tiny.

**Figure 3:**
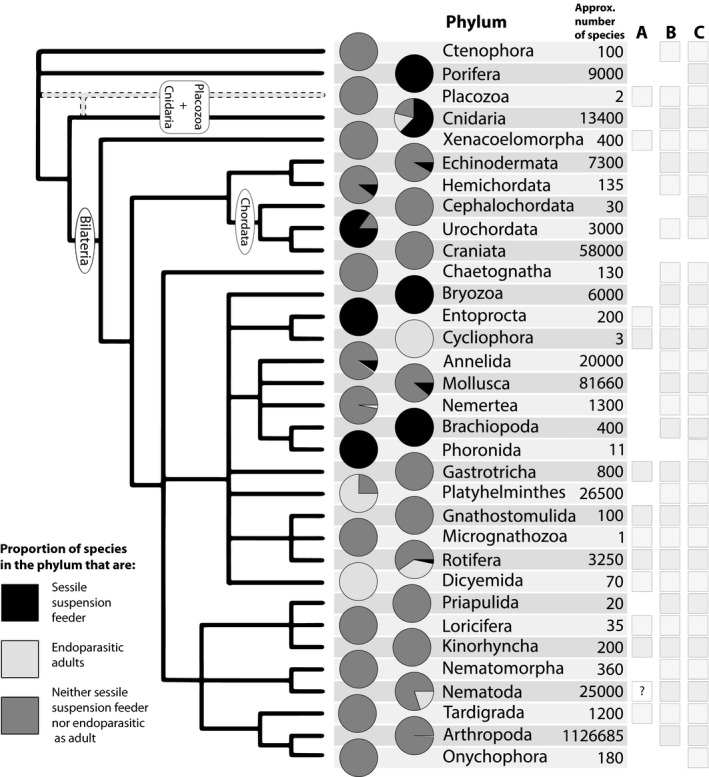
The major animal phyla, their species richness, and the proportion of taxa that are sessile suspension feeders (light gray), endoparasitic as adults (black), or neither (i.e., often predatory or deposit feeding; medium gray). Letters in the right column refer to the following: (a) Lineage contains mostly meiofaunal species (less than 2.1 mm in length); (b) contains at least one meiofaunal species; and (c) invertebrates. Various authors estimate that Nematoda contains vast number of undescribed species (e.g., (Creer et al., 2010)), and the balance of taxa may shift to being majority free‐living (often meiofaunal) or majority parasitic (sometimes meiofaunal, but this category also includes the largest Nematodes). Bilaterians that are sessile suspension feeders often display a secondary reduction in cephalization. Similarly, organisms that are endoparasitic as adults also often display profound morphological modifications, including reduction in ganglion size, loss of obvious segmentation in some pentastomids and endoparasitic copepods (Arthropoda), and the complete loss of a digestive tract in Cestodes (Platyhelminthes). For more information on how we generated the pie charts, see Appendix S1 & S3. We hope we have sufficiently irked at least one researcher to inspire a more thorough treatise on the evolution of sessile suspension feeders, endoparasites, or both! Phylogenetic tree modified with permission from C. Dunn after Dunn et al. (2014). Species counts from Brusca et al. (2016) except where noted in S3

Invertebrate animals are an intriguing group of organisms for teaching evolution and increasing the public's understanding of what evolution produces (Figures 2 and 3). First, studying animals outside of vertebrates is an opportunity to reconstruct one's own place within evolutionary history, resetting an entrenched framework with humans at the top of a ladder of progress. Second, invertebrates comprise more than 95% of animal diversity (Brusca et al., 2016), and so teaching about them is an opportunity to expose students to novel material while simultaneously reinforcing key evolutionary concepts. An invertebrate approach serves not only evolution courses, but also diversity of life, and introductory biology courses. Third, whereas macroevolution education increasingly emphasizes debunking evolution myths through tree thinking (Catley et al., 2012), teaching about invertebrate evolution takes tree thinking out of the conceptual realm of phylogenetics and applies tree‐based logic to real patterns and examples among animals throughout the entire animal phylogeny. Fourth, invertebrates are weird (Figure 2). We think that this is something to embrace rather than shy away from, since bizarre, sometimes disgusting, examples tend to evoke emotion and therefore improve memory and recall (Anderson et al., 2006; Cahill & McGaugh, 1998). Students engage with content that provokes them.

Our fellow invertebrate zoologists might balk at our deliberately derogatory language in the title. Is not every intestinal parasite beautiful and wonderful in its own way? Yes. And we hope our colleagues read this as the love letter to spineless metazoans that it is. We think it more expedient to affirm some students' initial reactions to metazoan diversity as a jumping‐off point. An important goal of ours is to point out the idea that evolution can result in forms that an average person might find neither beautiful nor wonderful. We think the deliberately derogatory words in the title serve an important purpose that would be lost if we opted for more objective language. Are dicyemids "smart," because they have "figured out" how to live within their hosts? No. Despite the poetic utility of likening evolution by natural selection to problem‐solving by neurological processes, dicyemids have zero neurons (Furuya et al., 2004), and so are lacking in intelligence. Dicyemids are stupid. Octopuses are smart (Amodio et al., 2019). This paper is aimed at celebrating the "stupid" and the superficially revolting animals. If we accept that the average person would not think a highly magnified picture of a soil mite is beautiful, how can we communicate the message that evolution has given us many more of these than birds of paradise? If someone believes that everything that has evolved is beautiful, we do not think this essay will change their mind—nor would we want it to.

Below, we illustrate specific ways in which the intuitive appeal of teleology can be mitigated with invertebrate biology combined with a healthy dose of tree thinking. The examples, explanations, and figures that follow are intended for use in the classroom and public outreach, and we hope they are useful to everyone who communicates about the way evolution works. One of us (RJR) teaches invertebrate zoology at SUNY‐ESF, so she has used many of these examples in the classroom. Both JCM and RJR have taught the general evolution course at SUNY‐ESF, and we find at least some mention of the themes discussed below is important as a conceptual counterpoint when talking about sexual selection, or the evolution of whales or hominins. We certainly think that traditional, charismatic systems are still useful in the classroom (e.g., we still teach the “typical” examples in Table 1), but we hope what follows inspires educators to occasionally stray from the paths more trodden with interjections of “… but also…” Better yet, the examples that follow could be used by students to develop their own short lessons contrasting an example from this paper with a “typical” example from Table 1 to teach their classmates and instructor about the many trajectories evolution can take.

## 2 MAJOR IMPRESSIONS AND EVOLUTIONARY LESSONS FROM THE TREE OF ANIMAL LIFE

Most major animal lineages are invertebrates (Figure 3, column C). Vertebrates versus invertebrates are the way most people organize animals, but this dichotomy is erroneous since there is no monophyletic clade (i.e., a group including an ancestor and all its descendants) called “invertebrates.” There are simply animals (Metazoa), with the vertebrates as the single lineage of backboned animals within the Chordata (the “Craniata” branch on the Chordata clade, Figure 3). Although chordates are often represented as the top of a ladder‐like sequence, tree thinking reveals that Chordata is not the end of the line in a straight progression from sponge to ape. Rather, each branching point or node can be spun around, so that the Onychophora is just as easily the “end” of the tree (Figures 1 and 3). Today's jellyfish species are not ancestral forms, but merely extant, very recently evolved relatives and representatives of early animals. Importantly, neither chordates nor arthropods are “more‐evolved” or “less‐evolved” since they have each had the same amount of time to evolve since they split at their last common ancestor represented by that node (Figure 1). Modern sponges, though commonly thought of as “primitive,” are just as far away from the base of the tree as chordates or arthropods are (Figure 3). They also have true tissues and can even “sneeze” (Leys, 2015).

Representatives of nearly every major lineage of animals (most of which are at least partially marine) appeared in the fossil record around the same time, 541–520 Ma (earliest animals potentially evolved as early as 800 Ma (Dunn et al., 2014)). During this time, extinction, not just speciation, has played an important role in shaping animal diversification patterns. It would likely surprise most people that the seemingly destructive, “antiprogress” force of extinction has been instrumental in shaping our modern fauna. For example, all living sea urchins and sand dollars (Echinodermata: Echinoidea) are descended from a few taxa that just happened to make it through the biggest mass extinction known, the End‐Permian (252 Ma) (Erwin, 1994, 2006; Koch & Thompson, 2020). We will never know whether these surviving species were actually better adapted than all the others; random chance is just as likely (Erwin, 2006). And some lineages that went extinct might be considered just as successful, if not more so, than groups that are extant. For example, trilobites, extinct arthropods, lived for nearly 300 Ma (Brusca et al., 2016), about 140 Ma longer than placental mammals.

Upon this backdrop of the animal tree and the more than 541 million years of speciation and extinction that shaped it, we still can fall into the trap of intuition when we see that so‐called “lower” animals are not only early‐diverging lineages, but possess morphologies that harken to less complex status. Therefore, it seems intuitive to use the tree to polarize, for example, “things with legs” and “things without legs” as more or less complex, respectively. But streamlined body form does not indicate lower status in evolution (O’Malley et al., 2016; Ryan & Chiodin, 2015). In this paper, we will demonstrate that body form simplicity and reductions are common throughout animal phylogeny (even within the “things with legs” category), and not just among early‐diverging lineages.

## 3 SIMPLICITY AND EVOLUTIONARY REVERSALS: CEPHALIZATION AND NERVOUS SYSTEMS

We can use an evolutionary tree to understand the pattern of certain adaptations across all of animals and the gains or losses of those traits over time. Is there evidence for progress? One of the most obvious and seemingly most useful animal adaptations is a head. Cephalization, the possession of a concentrated anterior nerve center, likely evolved alongside a motile foraging lifestyle, in association with the evolution of bilateral symmetry (see Bilateria, Figure 3). Animals possess a variety of head styles, but most include sensory structures such as ocelli (eyes), whiskers, and gravity‐sensing statocysts, a mouth, as well as concentrations of neurons into ganglia, or—when obvious enough—brains. Because our most distant relatives within the Metazoa lack heads, we tend to associate lack of cephalization with “lower” seemingly less successful animals. But, two points undercut this notion.

First, lack of a head is not debilitating to jellyfish. Their relatively diffuse distribution of nerves and ability to collect information from all sides of their bodies allows them to exploit food sources in their pelagic, boundary‐free environment, and likely contributed to their diversity (and increasing abundance (Brotz et al., 2012)) across the globe. Their cnidarian cousins, the corals, can build massive structures such as reefs, islands, and atolls that persist across entire geological time periods. There is also overlap between cnidarian nervous system complexity and that of some seemingly “higher” bilaterians: Cnidarian planula larvae have at least 102 neurons (Nakanishi et al., 2012), whereas male interstitial annelids *Dinophilus gyrociliatus* (Bilateria) have only 68 neurons (Minelli, 2009) and orthonectid annelids may have 10 or fewer (Schiffer et al., 2018; Slyusarev & Starunov, 2016). Other seemingly “simple” headless body plans, such as that of the sponges, belie unexpected complexity. Sponges possess epithelia, coordinate their movements, and sense the world, all without striated muscle and nerves. Their ecological success and the mode through which they achieve it is likely closely tied to their status as filtration powerhouses. Sponges also possess many genes in common with other seemingly more “complex” animals (Dunn et al., 2015).

Second, head loss and nervous system reduction is rampant among Bilateria. For example, the evolution of parasitism is associated with a severe reduction in nervous system complexity. At least one cestode (Figure 2f: Platyhelminthes) apparently lacks ganglia entirely (Lyngdoh & Tandon, 1992). In contrast, their free‐living relatives have brains with at least 8,000 neurons (Agata, 2008). Invertebrates range from having zero nerves in Porifera, the tiny amorphous Placozoa (Brusca et al., 2016), and the endoparasitic dicyemids (Furuya et al., 2004; Schiffer et al., 2018), to having approximately half a billion neurons, in the genus *Octopus* (Young, 1963). Between these extremes, a variety of evolutionary trajectories have played out.

There are also many examples of secondary derivation of headlessness, or substantially reduced brains, among sessile suspension feeding animals (Figure 3). Barnacles (Arthropoda) are sessile suspension feeders that have brains with fewer than 200 neurons (Schnapp & Stuart, 1983). Among molluscs, bivalves have nonconcentrated nervous systems and lack a head, despite the fact that their ancestors were almost certainly cephalized (Brusca et al., 2016). Generally speaking, bivalves' anterior soft parts are palps that assist in sorting particles of food that enter their bodies via ciliary currents on their gills during suspension feeding (Brusca et al., 2016). When you compare a bivalve sitting on or in the sea floor to a predatory squid, a cephalized cousin of bivalves, it would seem that the squid is the evolutionary winner, with bivalves relegated to mucky habitats. But most bivalves are headlessly filtering large quantities of water with their gills, exploiting an omnipresent food source from a secure location, a lifestyle ideally suited to the vast marine realm, 71% of Earth's surface area (e.g., one oyster: 6.8 L per hr (Riisgård, 1988)). While doing so, bivalves have managed to diversify into 9,200 living species in every aquatic habitat on the planet (Figure 3) (Brusca et al., 2016). They are also among the oldest noncolonial animals on Earth: Ming the clam (*Arctica islandica*) lived to be 507 years old (Butler et al., 2013).

Big brains do not equal “success” or “progress” in evolution. Rather, just like any trait, form evolves to fit function: organisms that are sessile suspension feeders or endoparasites as adults tend to have reduced cephalization; miniaturized organisms could go either way (Figure 2d,e,f). Some of the most insignificantly sized arthropods are neurally gifted. Contrasting with mostly larger, suspension feeding barnacles and bivalves, some of the smallest insects have 5,000–10,000 neurons, likely related to flight and novel adaptations to miniaturization. They accomplish this feat by lysing the nuclei of most of their neurons at a late stage in pupal development (Polilov, 2015).

Recent phylogenetic studies have given us new perspectives on headless animals and the evolution of animal nervous systems. On the basis of their morphologies and apparent relative complexity, earliest evolving lineages were suspected to have evolved in the following seemingly progressive order: Porifera–Placozoa–Cnidaria–Ctenophora. This traditional view has been supported by previous phylogenetic hypotheses and reinforced by morphology. But recent phylogenomic data support a radical view: ctenophores anchor the tree of animals, followed by Porifera (Figure 2d), Placozoa, and Cnidaria (Dunn et al., 2008; Dunn et al., 2014; but see Philippe et al., 2009; Laumer et al., 2018). The idea that seemingly simpler animals diverged from our lineage (the Bilateria) after ctenophores cuts at the core of any notion of evolutionary progress. One major sticking point is the evolution of nerves, since nerves are traditionally thought to have evolved once, after sponges. If early ctenophores evolved first, followed by the nerve‐less Porifera and Placozoa, then either nerves evolved independently in ctenophores and the Cnidaria–Bilateria clade, or nerves were lost in Placozoa and/or Porifera (Dunn et al., 2014; Laumer et al., 2018; Ryan & Chiodin, 2015). For now, we are left with exciting uncertainty regarding the evolution of nerves given the implications of the ctenophores—first phylogenetic hypothesis (Dunn et al., 2015).

## 4 ENDLESS TINY FORMS

If we looked closely at most major lineages of animals, we would need a microscope (Figure 2g,h,i). Tiny forms are present in nearly all animal phyla—some groups have always been tiny and are solely composed of miniature members (Figure 3). Others are known for their macroscale species, but have a significant number of tiny species, some of which evolved from larger ancestors. The largest invertebrates are squids (*Architeuthis* and *Mesonychoteuthis*), but they share a phylum (Mollusca) with many tiny organisms such as *Ammonicera* and *Condylonucula*, a snail and a clam genus, respectively, that each contain at least one species that never gets larger than a half a millimeter. In North America, Europe, and New Zealand, the land snail fauna tends to be composed of 40% to nearly 100% of taxa that are 5 mm or less (Nekola et al., 2013). In one of the most thorough marine mollusc sampling efforts thus far, researchers found that median adult body size was 8 mm, with only 8% having an adult body size of more than 41 mm (Bouchet et al., 2002). The most numerous animals in the oceans are even smaller than the median mollusc: A copepodologist will tell you that copepods are likely the most abundant animals on Earth (Turner, 2004) and a nematologist will tell you it is nematodes (Creer et al., 2010). Nematodes may rival arthropods in diversity (Creer et al., 2010); thus, Arthropoda and Nematoda likely drive abundance‐based and species richness‐based trends in animal body size.

How do we explain the pattern of remaining or becoming small (Box 1)? Part of the answer lies in ecology. Body size pervades nearly every aspect of an organism's life, and therefore, it is an important route for adapting to different environments (Blanckenhorn, 2000). Two of the most neglected and weirdest, yet important habitats for tiny animals, are interstitial habitats (e.g., living between grains of sand) and within other animals or plants (e.g., parasites). Given the ubiquity of these outer and inner environments, it is no surprise that so many major lineages and species evolved there.

Interstitial habitats are a frontier for discovery (Balsamo et al., 2020; Creer et al., 2010; Rundell & Leander, 2010). These habitats include vast expanses of muds, sands, and shell hash at all marine and freshwater depths, and can include water films in habitats that seem terrestrial to macroorganisms like us. The animals that live between the particles, gleaning bacteria, and algae or hunting other tiny organisms are called meiofauna, and range in size from approximately 60 µm to 2 mm. Nearly every major animal lineage includes meiofaunal species, and twelve phyla are exclusively meiofaunal (Rundell & Leander, 2010). Tiny species often share adaptations to the interstitial lifestyle, for example, sticky toes for adhering to sand grains, or body armor to resist crushing by constantly shifting sand grain boulders. A fairly new phylum is the Loricifera (discovered in 1983 (Kristensen, 1983)), whose species live solely in marine sediment feeding with a structure called an introvert. Sandy searches for meiofauna have revealed not just new species but curious innovations that belie their reduced size. Some tardigrade species perform a courtship ritual, where the male strokes the female with his cirri, and once the female lays her eggs on a sand grain, he spreads his sperm on them. Like some other meiofaunal groups, tardigrades grow in size not by adding more cells but by increasing the size of their cells (eutely (Brusca et al., 2016)).

Some miniature insect species lay their eggs inside other insects' eggs, and the larvae slowly eat their crib, ultimately killing the egg before it can develop. Symbiotic relationships in which the host organism (or egg) is killed are parasitoid relationships: more lethal than some forms of parasitism and more prolonged than regular predation. There are probably more than 100,000 insects that are parasitoids, mostly in the hymenopteran superfamily Chalcidoidea (Forbes et al., 2018; Heraty et al., 2013). The chalcidoid families Trichogrammatidae and Mymaridae each contain several species with adults that are smaller than 300 µm, including taxa with whimsical names such as *Tinkerbella* and *Kikiki* (Polilov, 2015). The only other insects known to approach this size are members of the featherwing beetle family, Ptiliidae. Ptiliids, trichogrammatids, and mymarids share some derived characteristics that accompany their miniaturization, like the condition of having wings with fine fringes around them (ptiloptery). All three families also lack a heart at some stage of development when most insects have a heart: The wasps Trichogrammatidae and Mymaridae often lack hearts as larvae; ptiliid beetles lack hearts as adults. Extreme miniaturization often leads to morphological simplification, as in the endoparasitic dicyemids (a group of animals related to annelids, molluscs, and flatworms that parasitize cephalopods), which can have fewer than 40 total cells, and lack nerves (Furuya et al., 2004; Schiffer et al., 2018). However, insects are able to buck the trend of reduced nervous system size in response to miniaturization, and retain fairly complex nervous systems of thousands of neurons (as opposed to the hundreds or fewer retained by some meiofauna).

Perhaps the most spectacular examples of miniaturization occur in taxa that exhibit sexual dimorphism. Examples of miniaturized males in sexually dimorphic invertebrates include *Nephila* spiders, *Osedax* “zombie worms” (Annelida), and the blanket octopus *Tremoctopus violaceus* (Mollusca) (Rouse et al., 2008; Vollrath, 1998). In one of the most extreme examples, *Dinophilus gyrociliatus* (Annelida), mentioned earlier: The male is only 50 µm, and so is one of the smallest animals known. Arguably, the most extreme is what are almost unicellular males in the scale insect *Icerya purchasi* (Arthropoda). This species, and perhaps others related to it, have unipotent sperm present in all observed females. These sperm can undergo cell division and fertilize the female's eggs inside her body. During or immediately after fertilization, other sperm associate with the egg before oviposition and live commensally in the developing female. There does seem to be loosely organized spermatogenic tissue in the female, but it seems to be descended from a line of sperm that can persist in the female's body, then fertilize her eggs, and be passed down transovarially (Gardner & Ross, 2011; Normark, 2003; Royer, 1975). This bizarre permutation on hermaphroditism is difficult to categorize, but from a functional perspective might represent the most profound miniaturization event in animal evolution: a state that approaches secondarily evolved unicellularity.

Other twigs of the metazoan tree that approach unicellularity are transmissible cancers (Chen et al., 2015; Duesberg et al., 2011; Ujvari et al., 2016, 2017). Although capable of growing into aggregations of cells (just like any cancer), transmissible cancers have an evolutionary trajectory of their own and bypass the typical metazoan stages of embryonic development and cellular differentiation. The most famous transmissible cancers are from Tasmanian devils and domestic dogs, but there are several known lineages that impact bivalves, alternately infecting and growing in the host or breaking off as smaller “individuals” that can be taken up through suspension feeding. It seems likely that more of these transmissible cancers await discovery, especially in other sessile marine suspension feeders (Murchison, 2016; Ujvari et al., 2017). Controversially (and problematically (Weasel, 2004)), Van Valen et al. argued that the famous HeLa cells could be considered a new species (Strathmann, 1991; Van Valen & Maiorana, 1991). Although we do not endorse this view, many of their arguments were prescient to a macroevolutionary understanding of transmissible cancers as extremely simplified metazoan lineages.

Box 1 **Copious Evolutionary Trends**

Cope's rule has a long history in paleontology as an appealing hypothesis to test (Newell, 1949). Recently, Smith et al. (2016) defined Cope's rule as: “An empirical pattern of lineages evolving larger body sizes over time, in its strictest sense resulting from size increase within lineages.” The authors then say: “Recent work (Heim et al., 2015) suggests Cope's rule may be widely supported over many taxa and broad timescales.” Elsewhere the authors write: “All extant animal groups except insects, reptiles, and ostracods have achieved larger sizes today than earlier in Earth history.” We remind our readers that several other clades include much larger extinct members, including chelicerates, myriapods, and echinoderms. We challenge the reader to find an example of a species‐rich lineage of animals that has achieved a *smaller* size earlier in Earth's history than the current minimum adult body size (extinct brachiopods exceed both ends of the extant size range, but we cannot think of another diverse phylum or class that had a smaller minimum size in the past).

Heim et al. (2015) use a thorough dataset of fossil and extant taxa to show an increase in mean body size throughout the past ~540 Ma. However, through no fault of theirs, many meiofaunal taxa could not be included in their study. The authors' null models of minimum body size evolution predicted that the minimum observed animal body size could be as low as 10^–6^ mm^3^ by the present. This is actually close to the body size of the smallest animals: The smallest gastrotrich likely has a biovolume of ~6.67 × 10^–6^ (McClain & Boyer, 2009). Taxa smaller than the minimum body size (~10^−4^ mm) predicted by the model Heim et al. designed to simulate Cope's rule style of evolution have evolved in at least 5 animal phyla: the Gastrotricha, Rotifera, Nematoda, Annelida, and Arthropoda (McClain & Boyer, 2009). Through no fault of the authors, the most minute taxa have a sparse fossil record, and so could not have been included in the types of analyses they conducted. However, it is our view that Heim et al. have inadvertently overlooked data that would have changed their conclusions.

There are fascinating trends of body size increase in certain lineages during certain periods of Earth's history, given certain ecological contexts. But even the most charismatic extant giants, the mysticete whales, do not consistently exhibit Cope's rule: For 30 million years, the clade's body size evolution was indistinguishable from Brownian motion. It is only in the last 5 million years that their size has increased dramatically (Slater et al., 2017). Dinosaur macroevolutionary trends show evidence of multiple size optima (Benson et al., 2018). A meta‐analysis of microevolution studies of extant animals and plants did not support Cope's rule (Gotanda et al., 2015). We strongly affirm that evolutionary trends of gigantism are very interesting to study and teach, but we do not think that the “rule” part of Cope's rule is justified given the number of documented exceptions and the number of exceptions likely to exist in animals with poor fossil records. Clearly, there are compelling cases where gigantism has evolved as a result of a key innovation (zooxanthellae in giant clams, *Tridacna* (Stanley, 1973; Isozaki & Aljinović, 2009)) or exaptation to a particular niche. We reiterate Jablonski's point that “Large size is not universally advantageous, and multiple pressures operate on body size and taxon‐specific correlates that range from age at first reproduction to allometric morphologies.”(Jablonski, 1997).

## 5 MOST ICKY: EVOLUTION DEFIES GOOD TASTE

Many evolutionary biologists have emphasized that very beautiful organisms can evolve, but we think it is important to emphasize that evolution does not always lead to forms that the average person might immediately think are elegant or esthetically appealing. Thus far, we have focused on reversals and evolutionary lability, but certain groups may be ancestrally restricted in the ways they can develop: They are subject to constraint. This can sometimes result in strange, imperfect solutions to adaptive problems that other organisms have solved in seemingly more elegant ways (Figure 2a,b,c). For example, snails defecate on their heads. This seems to be the worst possible place to deposit your waste products: near your delicate sensory structures. Yet, snail species have defecated on their heads for 500 million years, diversifying into thousands of species, and show little sign of slowing down the anteriorly directed defecation train. Why do they insist? Part of the answer lies in torsion, a developmental pattern unique to gastropods, where the larval digestive tract twists into a figure eight. The anus that was once aimed straight back journeys to an anterior position, meaning the digestive tract can squish comfortably within a shell.

Just as most humans have an instinctive aversion to the thought of feces near their heads, many have an aversion to parasites. Parasitism has been discussed earlier as a means by which “regressive” evolution has occurred, often concurrently with the origination of evolutionary novelties: Cestode platyhelminths (tapeworms) possess interesting morphological innovations such as an anterior scolex that securely fastens them to the tissues of their interiorly facing home (Figure 2f), and some have evolved to function as colonial collections of clones continually fertilizing each other and sending off propagules (Świderski et al., 2007). Cestodes also lack one of the hallmarks of bilaterians: a gut. They have evolved structures that are endogenous to them, synapomorphies without analogue; simultaneously, they have lost brains and guts, and most likely a variety of sensory structures.

There are instances in evolution where gained and lost features combine in aggressively icky ways—perhaps nowhere more so than in the mating habits of bedbugs (Figure 2b; Insecta: Hemiptera: Cimicidae). Bedbugs practice “traumatic insemination,” and it can be somewhat mentally traumatic to learn about: reader beware. Traumatic insemination, in which males wound the females and inject sperm into her body cavity (or in which the male genitalia of a hermaphroditic invertebrate pierce the body wall of a conspecific), has independently evolved several times in different invertebrates (Lange et al., 2013; Tatarnic et al., 2014). Thus, bedbugs have gained novel penile morphology, but have lost some of the traits associated with female genitalia. This mating strategy is almost certainly costly to the female, but they tolerate it: Tatarnic et al. (2014) review cases indicating that accidental interspecific matings are often lethal to the female, but conspecifics have evolved a variety of means to deal with wounds and infection risk. In the Middle Eastern spider *Harpactea sadistica*, the male actually seems to envenomate the female first and *then* punctures her abdomen (or opisthosoma) with his accessory genitalia (the bulbi on his pedipalps) to inject sperm into her, often 6 or more times (Tatarnic et al., 2014). Evolving evermore intricate and variously penetrative male genitalia, however, is not a foregone conclusion. In cave‐dwelling *Neotrogla* insects, it is the females that have evolved a fantastic spined “gynosome” intromittent organ that functions as a spiny vacuum tube, anchoring to a male (which has a vagina‐like opening to facilitate penetration from the female) and allowing for ~40‐ to 70‐hr copulatory sessions (Yoshizawa et al., 2014).

Traumatic insemination is even more common among the more equitable hermaphroditic invertebrates (Lange et al., 2013). Some of the most evocative examples include polyclad flatworms that engage in competitive “penis fencing” and traumatic sperm insertion. The hermaphroditic flatworm that stabs first or more often ultimately increases its fitness payoff since it fathers more offspring and avoids the costs of embryo care (left to the partner). Flatworms' thin body construction and aquatic lifestyle may have initially driven evolution for reproductive streamlining (loss of female gonopore), but their reproductive evolution has since slithered in new and fascinating directions.

The evolution of sexual cannibalism also seems gratuitously graphic and unexpected. It has evolved a number of times (Elgar & Schneider, 2004), including as seemingly voluntary self‐sacrifice in male Australian red back spiders (*Latrodectus hasselti*), which essentially fling themselves toward the females' chelicerae during or after mating. Although both traumatic insemination and sexual cannibalism have likely been driven by sexual selection (Elgar & Schneider, 2004; Tatarnic et al., 2014), their existence also underscores the idea that evolution does not always lead to universally optimal (or esthetically appealing) solutions.

Richard Prum titles a chapter and a recurring theme of his book “Beauty Happens” (Prum, 2017). We share Prum's sense of wonder at the emergence of such extravagances as the dimorphic plumage of the great argus and many other birds, and we are intrigued by his account of the emergence of esthetic complexity. But we would add that icky happens, and it happens in forms that are more endless in their diversity. It is likely that each of the beautiful birds Prum has cherished throughout his career (and that we have reveled in learning about from him) is parasitized by lice, nematodes, flatworms, and any number of other icky things (Dobson et al., 2008).

## 6 CONCLUSIONS

“Could our impressions… arise as a psychological artifact of our preferential focus upon lineages that grow larger, while we ignore those that remain in stasis or get smaller—just as we focus on fishes, then dinosaurs, then mammoths, then humans, all the while ignoring the bacteria that have always dominated the diversity of life from the pinnacle of their unchanging mode throughout geological time?” (Gould, 2002).

The discovery of a few amorphous flatworms imperceptibly slinking along the floor of a deep sea canyon, and likened to dirty purple socks or *churros* (a Mexican dessert pastry), invigorated the study of members of the newly erected phylum Xenacoelomorpha (e.g., the long‐enigmatic *Xenoturbella*) (Rouse et al., 2016) (Figure 2a). Xenacoelomorphs seem morphologically conservative, but without a fossil record we cannot be sure. As we have argued above, evolutionary histories are replete with seemingly illogical reversals, and tiny, sometimes repugnant animals. But among these oddities, novelties, and seeming contradictions lies perhaps the most unnerving evidence refuting progress: the commonness of no morphological change at all, or stasis. For example, recently discovered loriciferan fossils seem morphologically nearly identical to modern loriciferans (Cerca et al., 2019; Harvey & Butterfield, 2017). In analyses of 251 traits measured across geological time, from organisms with relatively complete fossil records (e.g., tiny bean‐shaped crustaceans called ostracodes, as well as molluscs and trilobites), only 5% showed a directional trend, with the remainder divided equally between stasis and random change (Hunt, 2007). In a more recent study with a similar dataset, the authors found evidence of stasis in 30%–45% of lineages, depending on how stringently the models were applied, and evidence of directional trends in about 10% of lineages (Voje et al., 2018).

Both studies are consistent with the idea that stasis and random walks are important evolutionary patterns that might be more common than directional evolution (at least at time scales from 0 to 20 my). The examples discussed above: The evolution of simplification in placozoans and orthonectids; the spectacular miniaturization of interstitial meiofauna and insect egg‐parasitoid wasps; the strange and icky mating behaviors of bedbugs and flatworms; the steadfast efficiency of suspension feeders; and the myriad bizarre morphologies of endoparasites, along with the records of stasis and random walks from the fossil record, are evidence of macroevolutionary directions that would not be obvious from a cursory study of the evolution of whales, humans, and peacocks. There is grandeur in this view of metazoans, with their many traits, having evolved through a variety of evolutionary trajectories. While this planet has gone cycling on according to the laws of physics, endless forms most stupid, icky, and small have been, and are being, evolved.

## CONFLICT OF INTEREST

None declared.

## AUTHOR CONTRIBUTIONS

**Jesse Emrys Czekanski‐Moir:** Conceptualization (equal); investigation (equal); visualization (lead); writing–original draft (equal); writing–review and editing (equal). **Rebecca Janine Rundell:** Conceptualization (equal); data curation (equal); investigation (equal); supervision (lead); writing–original draft (equal); writing–review and editing (equal).

## Supporting information

Figure S1

Appendix S2

Appendix S1

Appendix S3

## Data Availability

No original data were collected for this manuscript. Species richness data used in Figure 3 can be found in the supplementary.
