# Supplementary figures and images for: Endless forms most stupid, icky, and small: The preponderance of noncharismatic invertebrates as integral to a biologically sound view of life
Source: Ecol Evol. 2020 Oct 15;10(23):12638–49. doi: 10.1002/ece3.6892 (PMC7713927; doi:10.1002/ece3.6892)

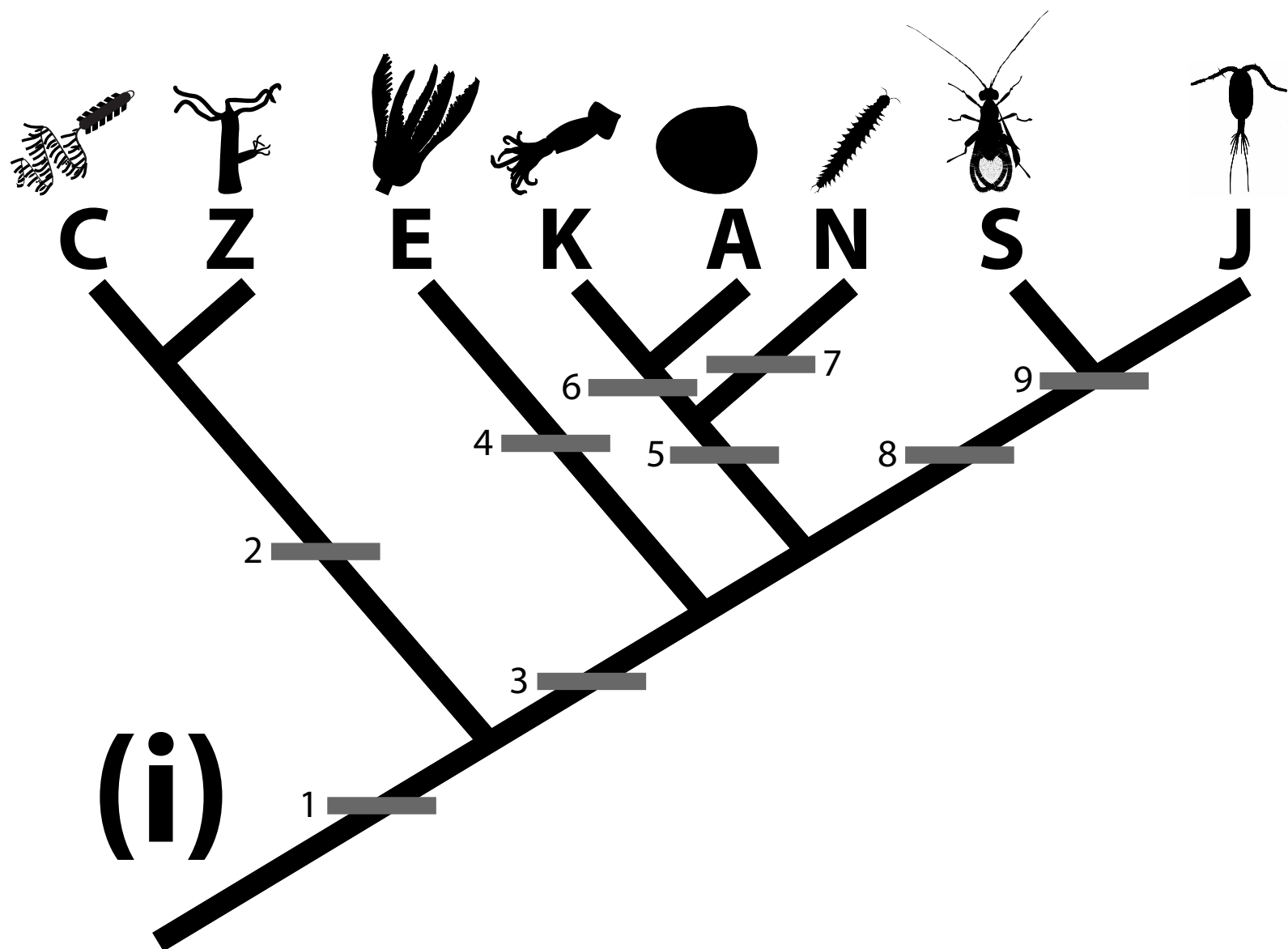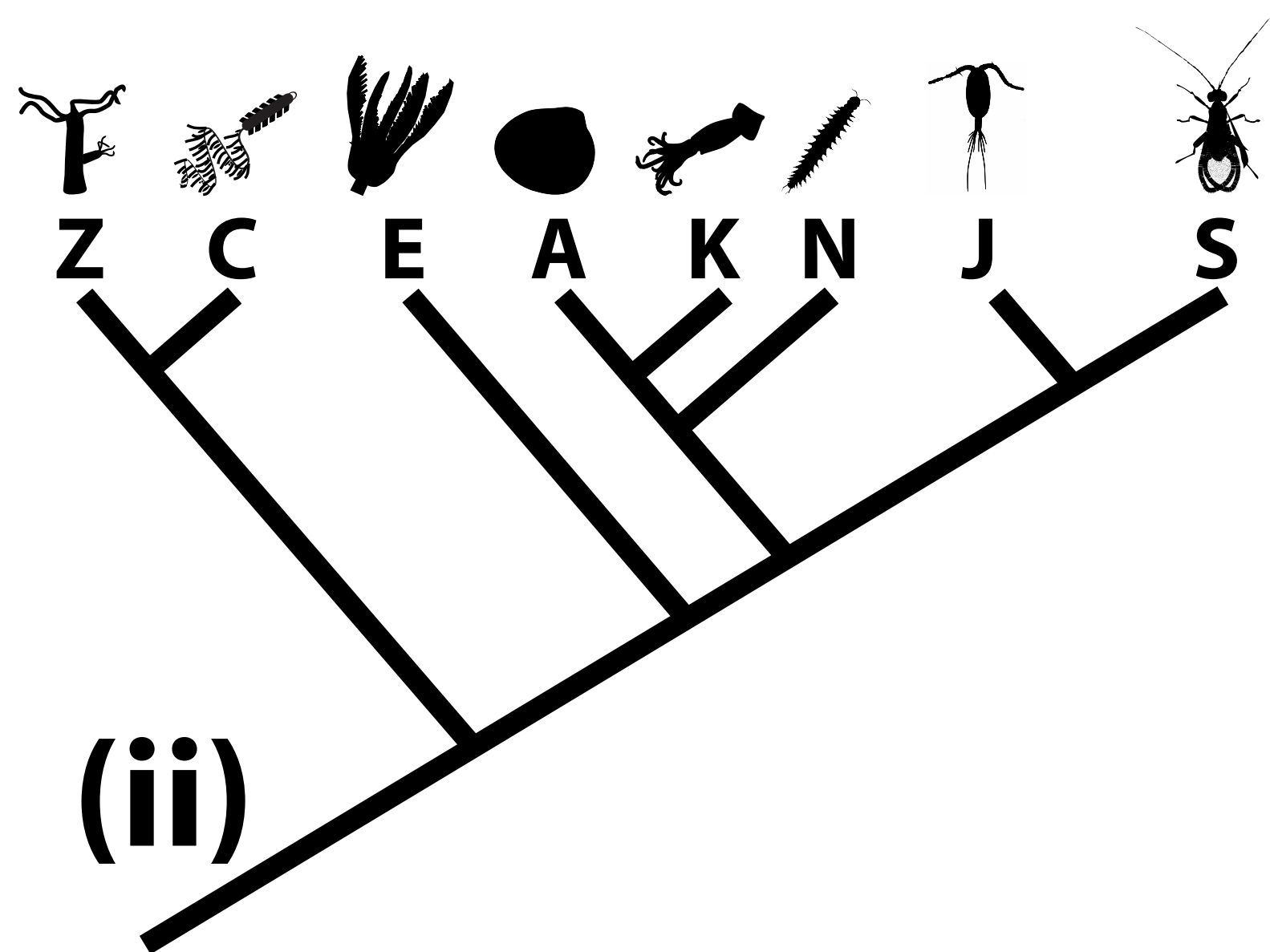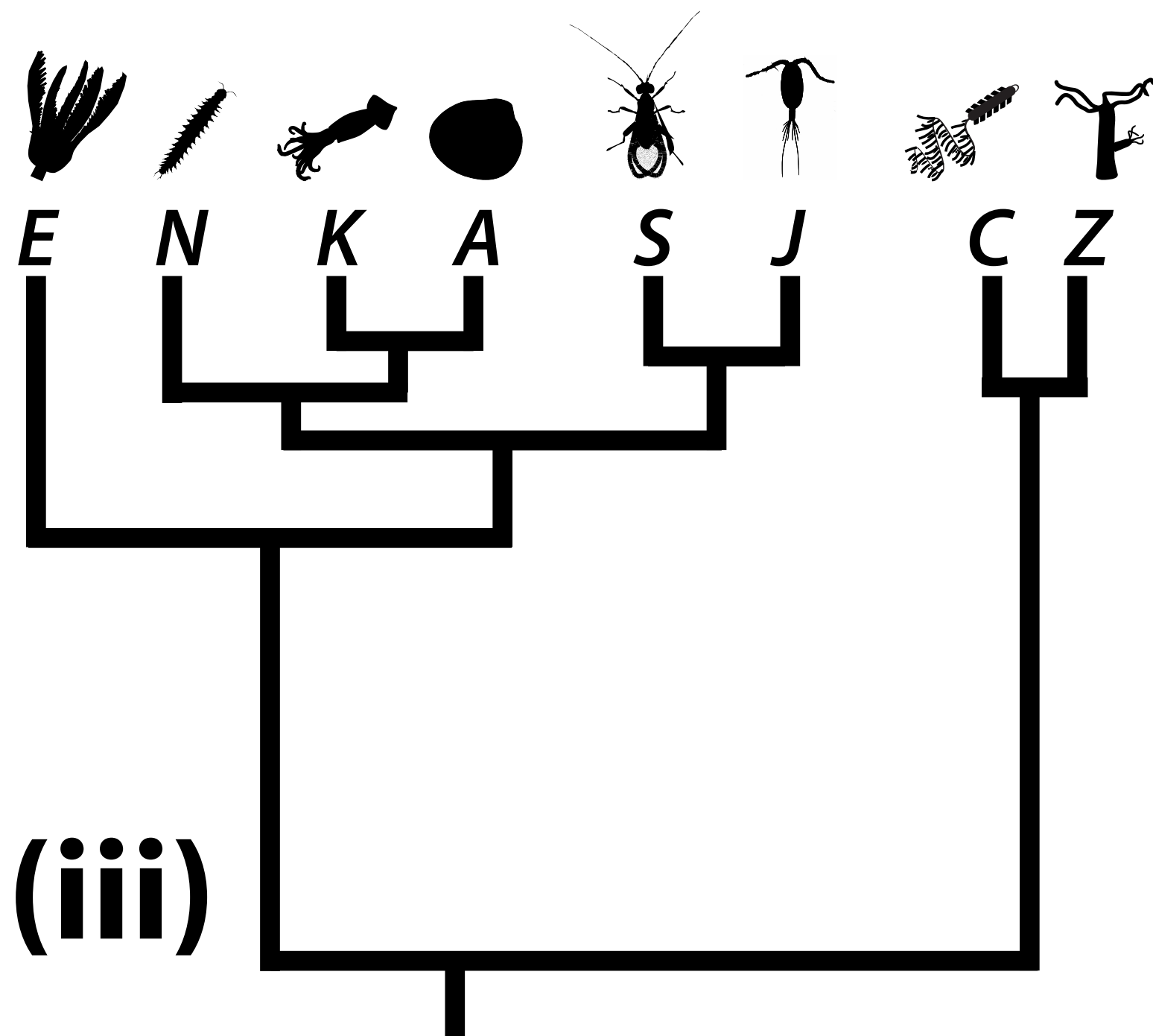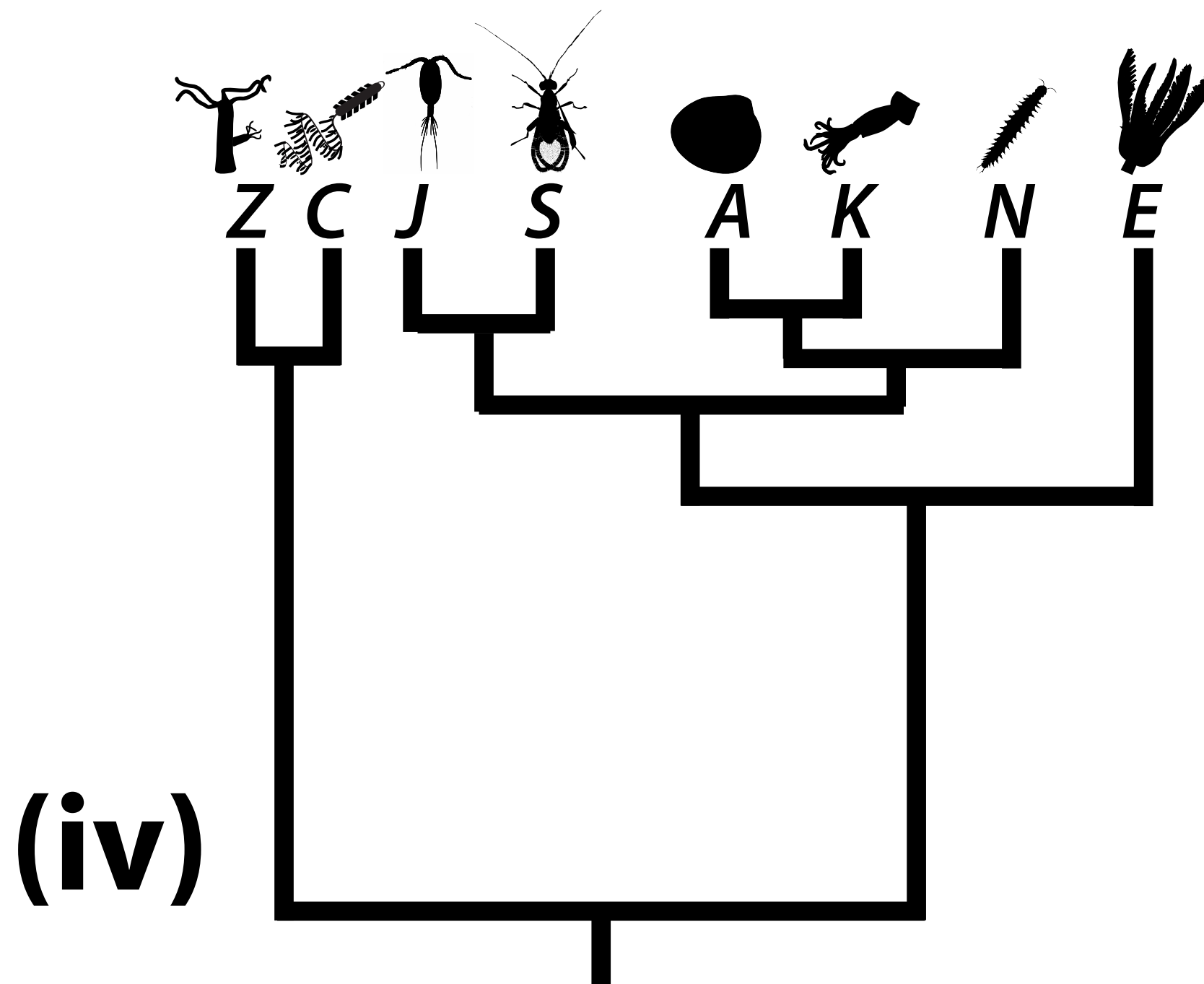

Supplement: Supplementary file 1 — Figure S1 [file ECE3-10-12638-s001.pdf]
