# Supplementary material for: Endless forms most stupid, icky, and small: The preponderance of noncharismatic invertebrates as integral to a biologically sound view of life
Source: Ecol Evol. 2020 Oct 15;10(23):12638–49. doi: 10.1002/ece3.6892 (PMC7713927; doi:10.1002/ece3.6892)
Supplement: Supplementary file 2 — Appendix S2 [file ECE3-10-12638-s002.docx]

**Supp 2: Further activity suggestions and information for Figure 1 (and Supplemental Figure 1)**

The numbered grey horizontal lines in the supplemental Figure 1 represent synapomorphies: evolved traits that are shared by the members of a clade (unless they are secondarily lost). One potential list of synapomorphies an instructor could use as a jumping-off point is:

1. Nerves

2. Nematocysts

3. Bilateral symmetry

4. Mutable collagenous tissue

5. Trochophore larvae

6. Mantle tissue

7. Serially homologous segments

8. Molting

9. Mandibles

A number of activities are possible with these trees, including:

- Ask the students to place the appropriate synapomorphies on the other trees.

- Ask students to use some of the phylogenetic resources below and draw approximately where taxa from figure 2 might fit into figure 1.

- Ask students to compare figures 1 and 3 from this manuscript, and ask them to think about which lineages might have secondarily reduced cephalization. What about the ecology of these organisms may have driven this shift?

- Ask students to look at other phylogenies and look up the names for the clades that include:

C & Z

K & A

K & A & N

J & S

- Emphasize that the trees are not scaled correctly to geologic time, and discuss how scientists can use fossils to scale the branch lengths without changing the topology. Students can work from a variety of primary literature sources to re-draw the branches so that they're scaled to evolutionary time. Although it is complicated, a good jumping-off point might be Figure 1 in[(Erwin et al. 2011)](https://paperpile.com/c/g306r4/oMWs)

Clade ((K, A), N) corresponds to the Spiralia: Lophotrochozoa (or just Trochozoa; see discussion in Giribet & Edgecombe 2020)

Clade (K, A) corresponds to Mollusca: Conchifera

For molluscs, [(Wanninger and Wollesen 2018)](https://paperpile.com/c/g306r4/rttq) provides a good overview phylogeny.

Clade (J, S) corresponds to Arthropoda: Pancrustacea (recall that insects are crustaceans in the same way that birds are reptiles [(Oakley et al. 2013)](https://paperpile.com/c/g306r4/NHrd)).

For Arthropods, figure 3 in  [(Giribet and Edgecombe 2019)](https://paperpile.com/c/g306r4/BH47) is a great overview. Students can use the timing of the last common ancestor between Copepoda and Insecta (~525Ma)

Clade (C,Z) corresponds to Cnidaria: Hydrozoa

For cnidarians, students can check out figure 2a in [(Khalturin et al. 2019)](https://paperpile.com/c/g306r4/tXGG). Although the siphonophore we're illustrating isn't on that phylogeny, students can use the divergence time between Physalia (another siphonophore like "C") and the Hydra genera (~390Ma, about 15my before Tiktaalik)

Erwin 2020 has a great overview of the potential timing of important developmental synapomorphies that could be mapped onto this phylogeny. Erwin’s Figure 1 can be used as a jumping off point for calibrating some of the divergence dates on the phylogeny [(Erwin 2020)](https://paperpile.com/c/g306r4/zhh5)

Other potentially useful resources include: [(Dunn et al. 2014; Droser et al. 2017)](https://paperpile.com/c/g306r4/IIij+a7pu)

For a nice overview of conceptual issues with teaching the animal phylogeny, see (Offner 2016)

Droser, M. L., L. G. Tarhan, and J. G. Gehling. 2017. The Rise of Animals in a Changing Environment: Global Ecological Innovation in the Late Ediacaran. , doi: 10.1146/annurev-earth-063016-015645. Annual Reviews.

Dunn, C. W., G. Giribet, G. D. Edgecombe, and A. Hejnol. 2014. Animal Phylogeny and Its Evolutionary Implications. Annu. Rev. Ecol. Evol. Syst. 45:371–395. Annual Reviews.

Erwin, D. H. 2020. The origin of animal body plans: a view from fossil evidence and the regulatory genome. Development 147.

Erwin, D. H., M. Laflamme, S. M. Tweedt, E. A. Sperling, D. Pisani, and K. J. Peterson. 2011. The Cambrian conundrum: early divergence and later ecological success in the early history of animals. Science 334:1091–1097.

Giribet, G., and G. D. Edgecombe. 2019. The Phylogeny and Evolutionary History of Arthropods. Curr. Biol. 29:R592–R602.

Khalturin, K., C. Shinzato, M. Khalturina, M. Hamada, M. Fujie, R. Koyanagi, M. Kanda, H. Goto, F. Anton-Erxleben, M. Toyokawa, S. Toshino, and N. Satoh. 2019. Medusozoan genomes inform the evolution of the jellyfish body plan. Nat Ecol Evol 3:811–822.

Oakley, T. H., J. M. Wolfe, A. R. Lindgren, and A. K. Zaharoff. 2013. Phylotranscriptomics to bring the understudied into the fold: monophyletic ostracoda, fossil placement, and pancrustacean phylogeny. Mol. Biol. Evol. 30:215–233.

Offner, S. 2016. Reconsidering Animal Phylogeny: Tree-Thinking. Am. Biol. Teach. 78:376–379.

Wanninger, A., and T. Wollesen. 2018. The evolution of molluscs. Biol. Rev. Camb. Philos. Soc., doi: 10.1111/brv.12439.

Taxonomy and copyright information for Figure 1 & Supplemental Figure 1

**A**: Mollusca; Bivalvia: unid Veneroida    Scott Hartman    Public Domain Dedication 1.0    <http://phylopic.org/image/9f6baa5c-7d56-46fd-b4d8-2de349898af0/>

**C**: Cnidaria: Hydrozoa: *Hydra*   Gareth Monger     <https://creativecommons.org/licenses/by/3.0/>    Creative Commons Attribution 3.0 Unported    <http://phylopic.org/image/16833d55-8147-4225-adec-874a328f1565/>

**E**: Echinodermata: unid Crinoidea    Lauren Sumner-Rooney    Public Domain Dedication 1.0    <http://phylopic.org/image/ab36081a-9d02-4d3a-9f18-feaddb440302/>

**J**: Arthropoda: Mandibulata: Insecta: Hymenoptera: *Leptopilina*     Kamil S Jaron    Public Domain Dedication 1.0    <http://phylopic.org/image/34f20053-ba76-4836-aaf5-9e85c56d4503/>

**K**: Mollusca: Cephalopoda: unid Decapodiformes (squids/cuttlefish)    Becky Barnes    <http://phylopic.org/image/443c7494-aa78-4a21-b2b4-cfa3639e1346/>

**N**: Annelida: Unid Polychaeta    Scott Hartman    Public Domain Dedication 1.0    <http://phylopic.org/image/2dee030d-9f6d-4bab-87c4-c46869839b30/>

**S**: Arthropoda: Mandibulata: Copepoda: *Erebonectes* Joanna Wolfe  Public Domain Dedication 1.0    <http://phylopic.org/image/c5dbd85a-c4be-4990-a369-c830ad23cb22/>

**Z**: Cnidaria: Hydrozoa: Siphonophore    No Copyright (Steve Haddock) Public Domain Mark 1.0    <http://phylopic.org/image/8a2b0cec-cb53-4ea0-ab30-7f840a47d7a6/>
