# Supplementary material for: Endless forms most stupid, icky, and small: The preponderance of noncharismatic invertebrates as integral to a biologically sound view of life
Source: Ecol Evol. 2020 Oct 15;10(23):12638–49. doi: 10.1002/ece3.6892 (PMC7713927; doi:10.1002/ece3.6892)
Supplement: Supplementary file 3 — Appendix S1 [file ECE3-10-12638-s003.docx]

Supplemental notes on the pie charts in figure 3

We only noted organisms with endoparasitic adults in this figure since these tend to be the most extensively modified. For example, there are likely to be more than 100,000 hymenopterans (Insecta: Arthopoda) with parasitoid larvae, but the adults are often active hunters, so they were not considered (but see discussion of extreme miniaturization in some parasitoid and free-living insects in this paper and in Polilov (2015 & 2016)). Similarly, every species in the phylum Nematomorpha is a parasitoid as a juvenile, but they are all free-living as adults. There are several candidates for endoparasites among craniate vertebrates, including fishes of the South American catfish families Stegophilinae and Trichomycteridae (the dreaded *candirú*), and the pearlfish (Carapidae), but these taxa are more close to the ‘micro-predator’ in the classification scheme of [(Lafferty & Kuris 2002)](https://paperpile.com/c/EqmIIW/BARkX), so we did not include any vertebrates as adult endoparasites, despite these taxa being frequently referred to as such. Analogous logic was applied to the gastropod families Pyramidellidae, Eulimidae, and Epitoniidae. Eulimids are sometimes found alongside pearlfish in the various cavities of holothuroideans, but also are quite capable of relocating to a new host, and so we assume that all of these families are technically micro-predators as well. Similarly, “sessile” is also a biological term with fuzzy boundaries. We did not include any holothuroideans as truly sessile, because as far as we are aware they can actively locomote (even if they often don’t). However, we did consider all of the Crinoidea to be sessile, even though many of them (even the stalked ones) can move, because their ancestor is presumed to have been truly sessile, and they retain much of the morphology from this heritage.
